# The Mystery of Color Photography

**Published:** 1881-09

**Authors:** 


### THE MYSTERY OF COLOR PHOTOGRAPHY.

A detailed account of the method of producing color photographs is given in the London *Photographic News*, from which it appears that two impressions are taken from the negative, the first being a weak impression in order to give the outlines for guiding the application of the coloring, and the second, after the colors have been applied, being an impression of sufficient strength to give the clear drawing, lights and shadows, and details of the picture. The first impression is colored with a hair pencil in vegetable colors, the various colors being laid on smoothly, flatly and lightly, without any regard to shading or softening off, but brighter than they are intended to be finally. The colors are specially prepared, and contain a large proportion of albumen, which enables them to be fixed by immersion in alcohol. This picture is again albuminized and sensitised, and undergoes a second exposure under the negative (which requires careful adjustment), a much stronger impression being obtained, and the picture is then firmly fixed, and can be enameled if desired.

